# Gorlin-Goltz Syndrome: A Case Report and Literature Review

**DOI:** 10.7759/cureus.3849

**Published:** 2019-01-08

**Authors:** Maha N Al-Jarboua, Abeer H Al-Husayni, Mohammed Al-Mgran, Ahmad F Al-Omar

**Affiliations:** 1 Dentistry, King Saud University, Riyadh, SAU; 2 Oral and Maxillofacial Surgery, King Saud University, Riyadh, SAU

**Keywords:** gorlin goltz syndrome, basal cell carcinoma, keratocystic odontogenic tumors

## Abstract

Gorlin-Goltz syndrome (GGS) is an infrequent multisystemic disease with an autosomal dominant inherited disorder characterized by the presence of multiple keratocystic odontogenic tumors (KCOT) in the jaws, multiple basal cell nevi carcinomas, and skeletal abnormalities. Early diagnosis of Gorlin-Goltz syndrome is essential as it may progress to aggressive basal cell carcinomas and neoplasias. Gorlin-Goltz syndrome has rarely been reported in Saudi Arabia. This article reports a case of a 13-year-old Saudi female patient with Gorlin-Goltz syndrome and includes an extensive literature review of the syndrome. To the extent of our knowledge, this is the first case reported by dentists in the Kingdom of Saudi Arabia.

## Introduction

Gorlin-Goltz syndrome (GGS), which is also well-known as nevoid basal cell carcinoma syndrome (NBCCS), is an autosomal dominant, rare multisystemic disease with a high degree of variable expressiveness and penetrance [1]. It is characterized by basal cell nevus, odontogenic keratocysts (KCOT), ectopic calcifications of the falx cerebri, and palmar and/or plantar pits [2]. Early diagnosis of GGS is important to reduce the severity of the complications, such as basal cell carcinomas and brain tumors, and to avoid the maxillofacial deformities related to the jaw cysts [3]. This paper presents a case of Gorlin-Goltz syndrome in a 13-year-old female patient. It also provides a literature review of Gorlin-Goltz syndrome.

## Case presentation

**Figure 1:**
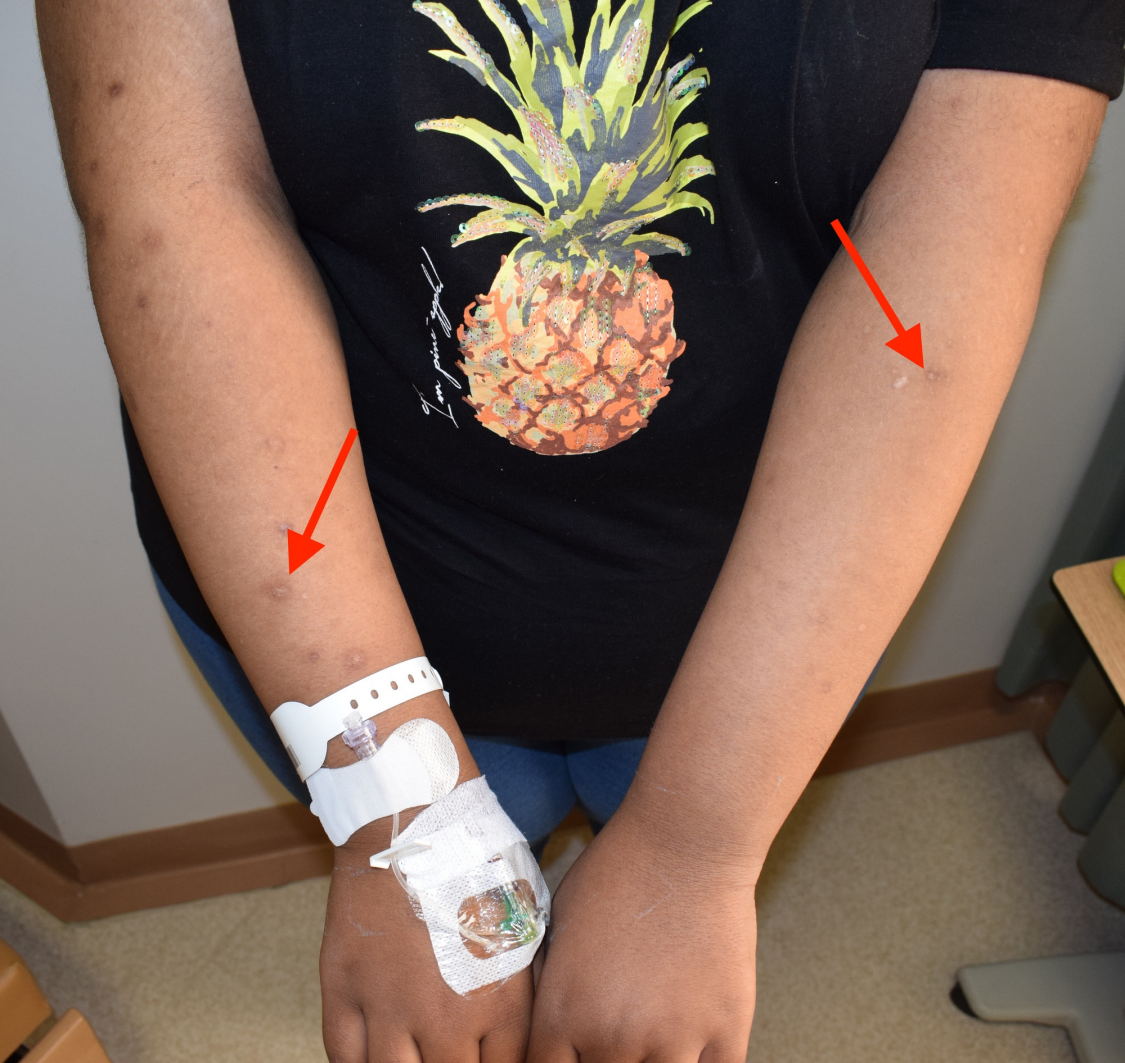
Multiple skin lesions (red arrows) on the patient's arms.

**Figure 2:**
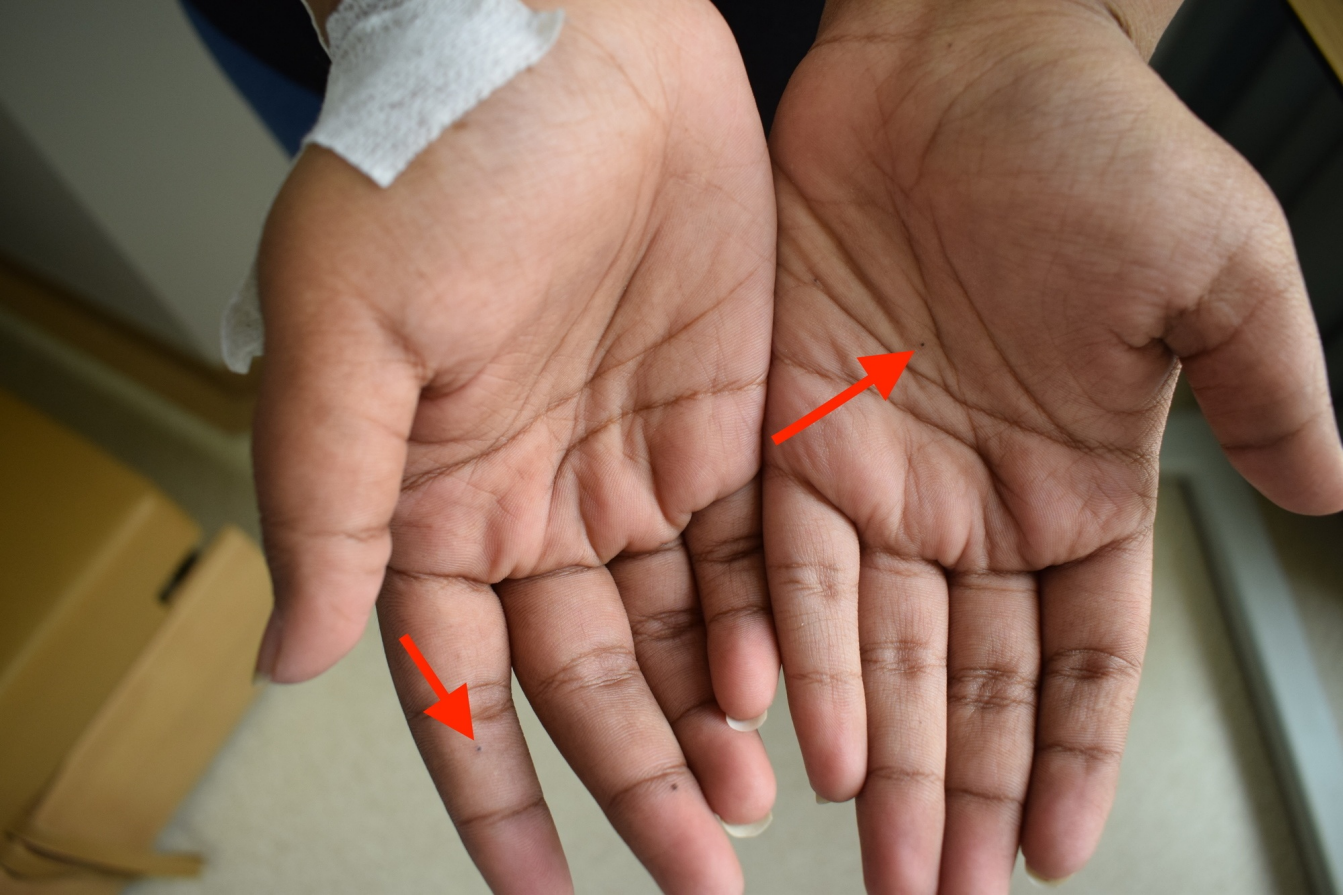
An examination of the patient revealed multiple palmar pits (red arrows).

**Figure 3:**
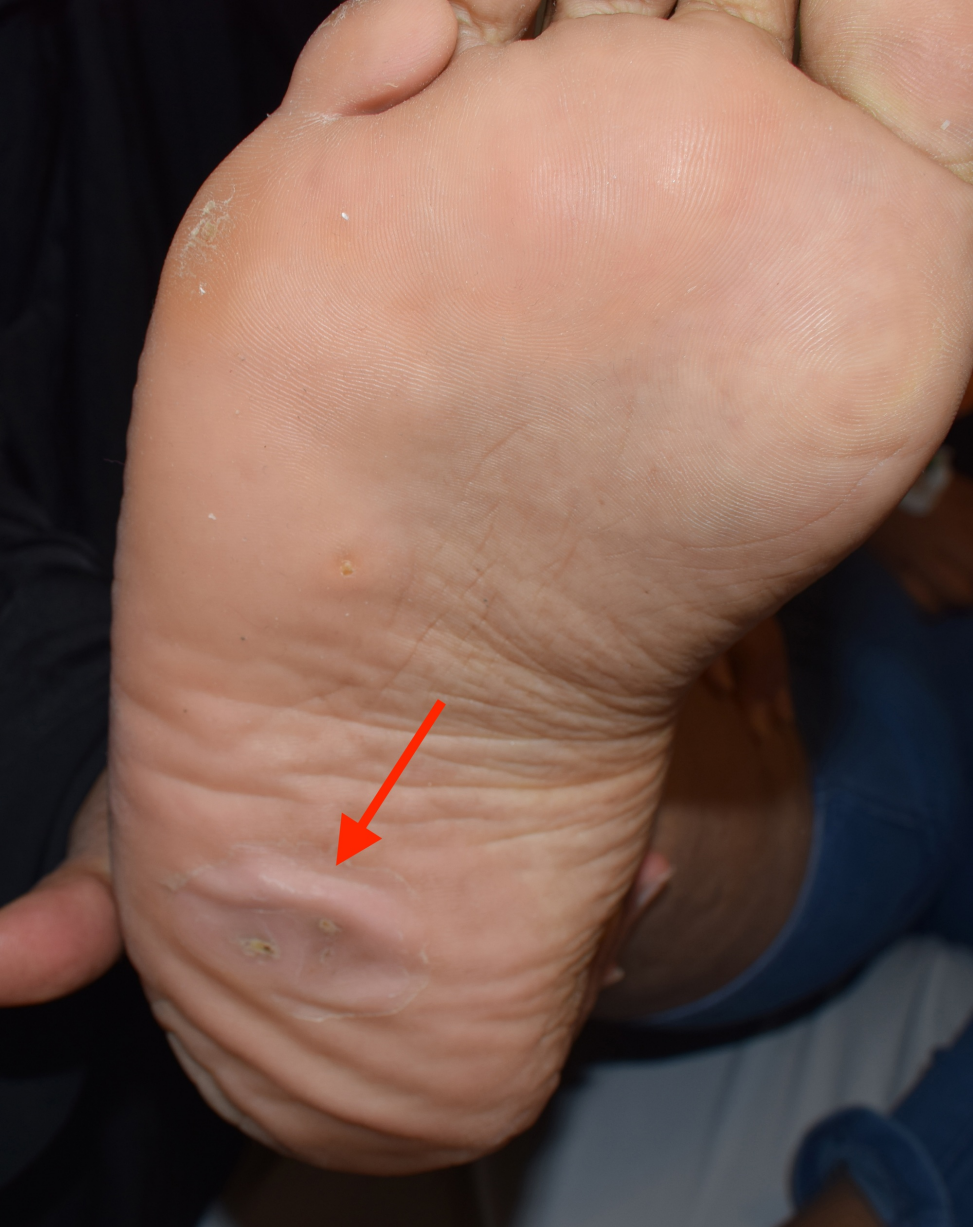
An examination of the patient revealed multiple plantar pits (red arrow).

A 13-year-old female patient presented to the Dental University Hospital at King Saud University, Riyadh, Saudi Arabia. The patient was referred by an orthodontist to restore her teeth before starting orthodontic treatment. Her weight was 125 kg and her height was 173 cm, which were abnormal for her age. The patient did not report any pain, discomfort or medical problems; however, the patient's response was late during the history taking. The patient had a history of successful repair of cleft lip and palate. Upon extra-oral examination, the frontal view showed facial asymmetry on the left side and flattening of the nasal bridge, increased intercanthal distance (35 centimeters) (orbital hypertelorism) and frontal bossing (54 centimeters), multiple skin lesions on her arms, and right foot and palmar/ plantar pits (Figures 1, 2, 3).

**Figure 4:**
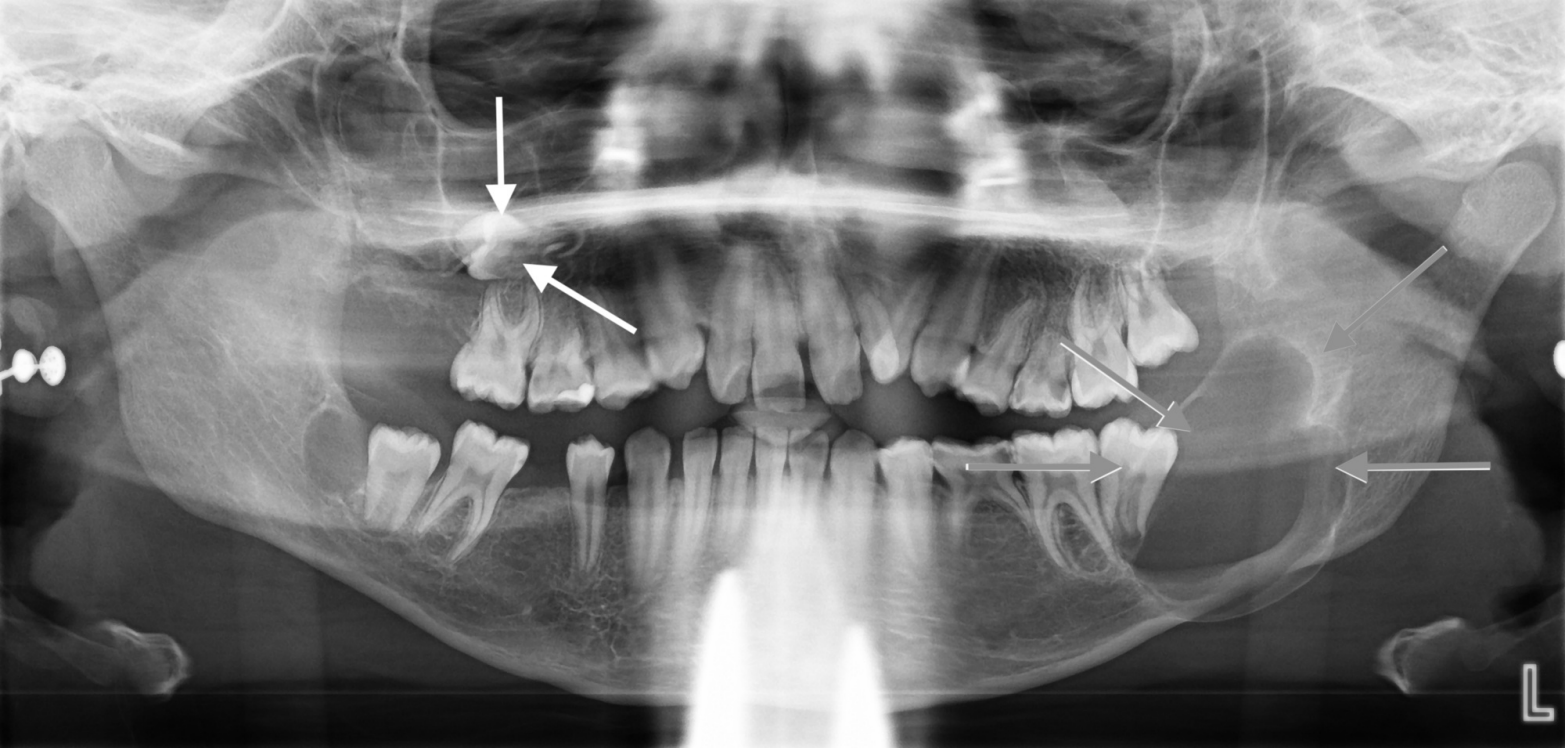
An orthopantomogram (OPG) shows the maxillary and mandibular lesions before the surgery. An orthopantomogram (OPG) shows a lesion associated with a partially erupted mandibular second molar tooth. The left mandibular lesion extended up to the ramus of the mandible (the gray arrows pointing down in the left side); moreover, the patient had another lesion associated with a horizontally impacted second premolar in the maxilla (the white arrows pointing up in the right side).

The intra-oral examination revealed multiple carious teeth, painless hard swelling in the left side of the mandible, and mild pain on percussion in all the left mandibular posterior teeth. An orthopantomogram (OPG) showed bilateral radiolucent lesions associated with a partially erupted mandibular second molar teeth. The left mandibular lesion was extended up to the ramus of the mandible; moreover, the patient had a horizontally impacted maxillary right second molar and congenitally missing second premolars and third molars (Figure 4).

**Figure 5:**
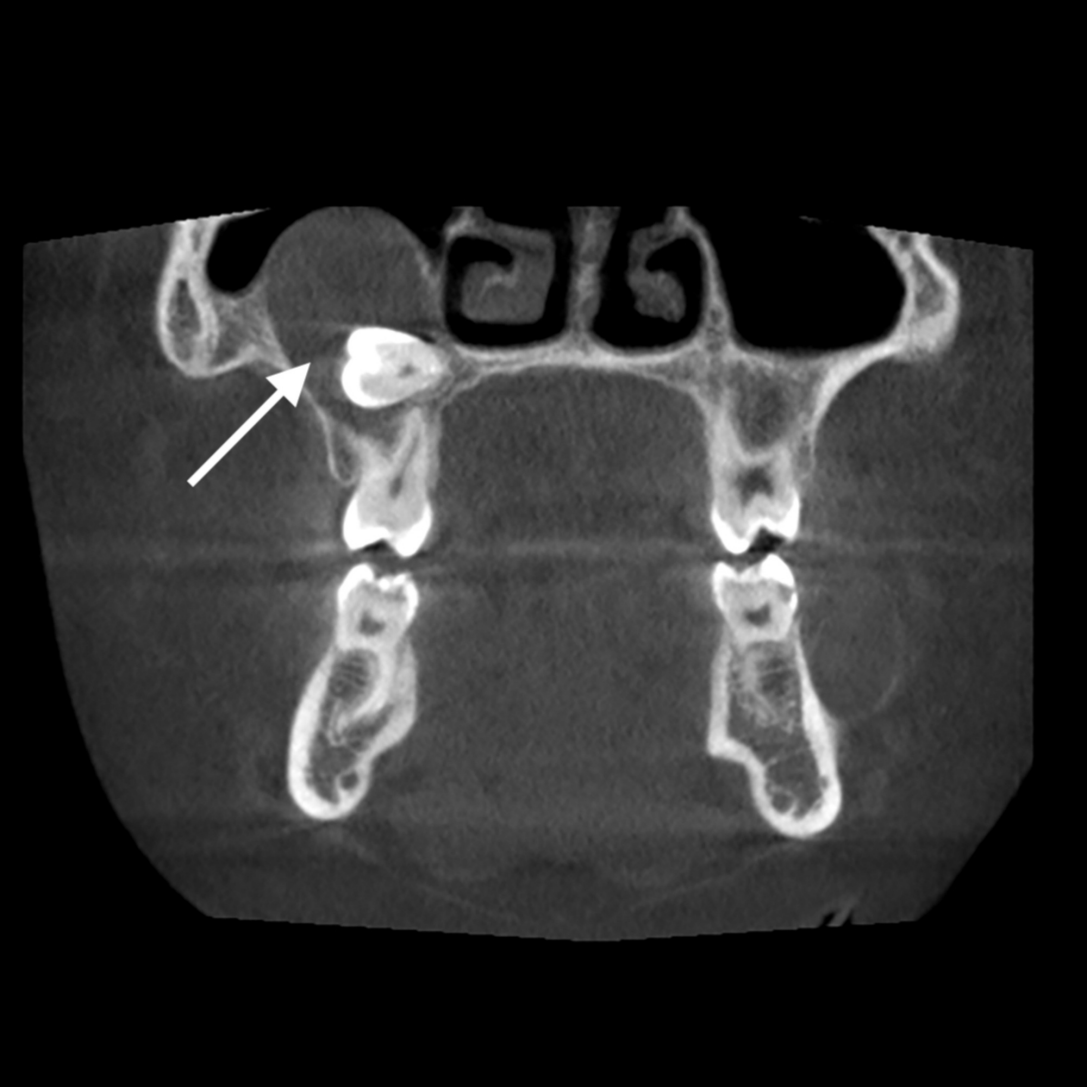
Cone beam computed tomography (CBCT) of another large lesion in the maxilla. Cone beam computed tomography (CBCT) revealed another large lesion associated with the impacted right maxillary second molar, which extended up to the right maxillary sinus (white arrow).

Cone beam computed tomography (CBCT) was requested for detailed radiological assessment of the mandibular lesions. Accidentally, CBCT revealed another large lesion associated with the impacted right maxillary second molar, which extended up to the right maxillary sinus. In addition, the lower left lesion was severely expanded in all directions (buccolingual, anteroposterior, and superoinferior), and perforations were noticed in the buccal and lingual borders of the mandible (Figure 5). A skull X-ray revealed calcification of the falx cerebra on the posteroanterior and lateral views.

**Figure 6:**
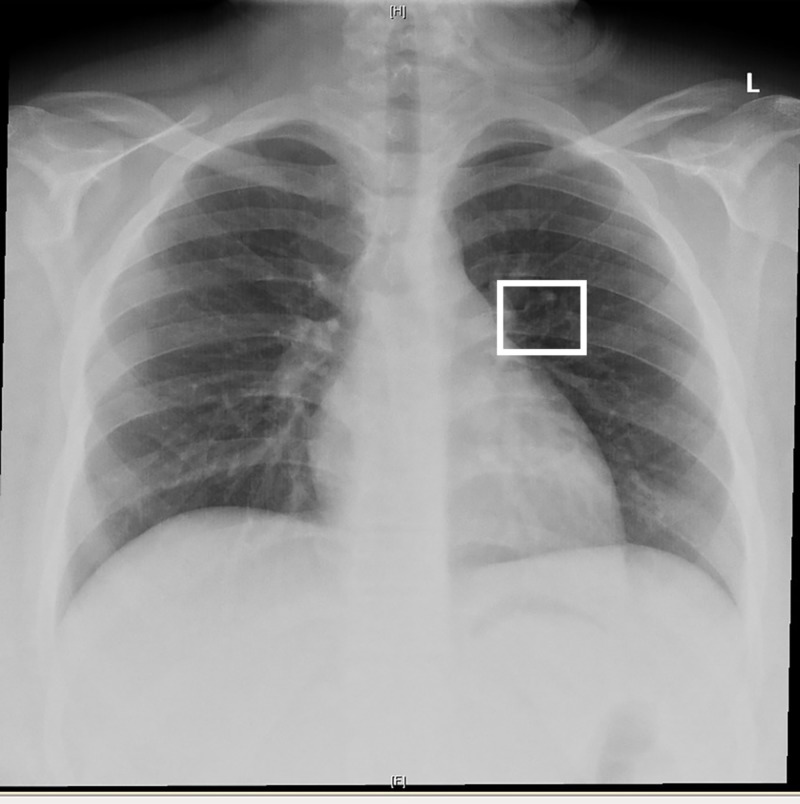
Anteroposterior view of chest X-ray. The anteroposterior view of a chest X-ray showed the presence of a bifid fifth rib on the left side (white box).

In addition, the anteroposterior view of a chest X-ray showed a bifid fifth rib on the left side (Figure 6).

A diagnosis of Gorlin-Goltz syndrome was made. Both maxillary and mandibular right cystic lesions of the jaws were enucleated surgically. Marsupialization was done for the mandibular left lesion and a biopsy was performed. The histopathological examination of the tissues showed KCOT of the right and left mandibular lesions and a dentigerous cyst of the maxillary right-side lesion. The patient was followed up every week after the surgery in the first month. The patient will then be followed-up once each month for six months. The tube will be removed after six months and then the patient will be followed up every six months.

**Figure 7:**
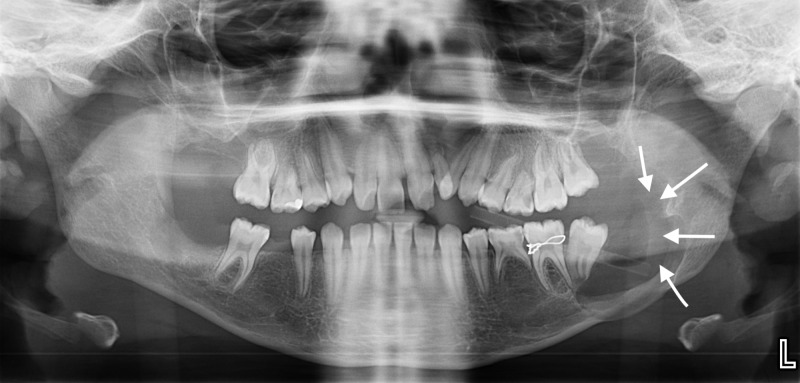
Orthopantomogram (OPG) after two months of follow-up. An orthopantomogram (OPG) after two months of follow-up shows improvement and bone deposition (white arrows).

After two months of follow-up, the marsupialized cyst showed improvement and bone deposition (Figure 7). Informed consent (written) was obtained from the patient and her parents. (The IRB approval number is E-18-3544).

## Discussion

Gorlin-Goltz Syndrome (GGS) is an infrequent genetic disease with variable manifestations. The prevalence of this syndrome ranges from 1 in 57,000 to 1 in 2,56,000 in the general population [3]. No sex predilection has been observed [4]. The first descriptions were made by Jarisch and White in 1894 and then detailed later by Gorlin and Goltz [5]. Researches have found that the cause of GGS was a tumor suppressor gene called Patched (PTCH), which is located in the 9q22.3 chromosome [6]. The mutation is transmitted from the parents to the sibling in an autosomal dominant way; however, Gorlin-Goltz syndrome can also arise from a spontaneous mutation (without any family history) in 35% to 50% of the cases [7]. Our patient in the presented case had no family history and Gorlin-Goltz syndrome was possible because of spontaneous mutation. The presence of two major or one major and two minor criteria is necessary for the diagnosis of GGS [8]. Evans et al. in 1993 established the major and minor criteria for the diagnosis of the syndrome, which was modified later by Kimonis et al. in 1997 (Table 1) [9, 10].

**Table 1:** Diagnostic criteria for Gorlin-Goltz syndrome (major and minor).

	Diagnostic Criteria	Present Case
Major criteria	Multiple basal cell carcinomas or single, which occur in patients under 20 years of age.	+
	KCOTs of the jaws (histologically confirmed).	+
	Plantar or palmar pits ( ≥three).	+
	Bilamellar calcification of the falx cerebri.	+
	Bifid, fused or markedly splayed ribs.	+
	First-degree relative with Gorlin-Goltz syndrome.	-
Minor criteria	Macrocephaly (adjusted for height).	+
	Congenital abnormalities: frontal bossing, cleft lip or palate, moderate or severe hypertelorism, coarse face.	+
	Other skeletal abnormalities: marked pectus deformity, marked syndactyly of the digits, Sprengel deformity.	-
	Radiographical abnormalities: modeling defects of the hands and feet or flame-shaped hands or feet, vertebral anomalies such as hemivertebrae, fusion or elongation of the vertebral bodies, bridging of the sella turcica.	-
	Ovarian fibroma.	-
	Medulloblastoma.	-

In the present case, the diagnosis of the Gorlin-Goltz syndrome was made due to the presence of five major criteria, which were multiple basal cell carcinomas (BCCs) present in a patient under 20 years of age, multiple KCOTs of the jaw bone, palmar or plantar pits, calcified falx cerebri, and bifid rib, and three minor criteria, which were cleft lip and palate, macrocephaly, and hypertelorism.

Multiple basal cell carcinomas are considered one of the most common characteristics of GGS, especially in the head and neck region [1]. BCCs may also appear on the trunk and limbs [9]. Another feature of GGS is the occurrence of multiple KCOTs of the jaw [11]. The occurrence of GGS-associated KCOTs is approximately a decade earlier than that of KCOTs not associated with the syndrome [12]; moreover, KCOTs are considered to be among the most frequent and consistent features of GGS. KCOTs are found in 65%-100% of the affected individuals [13]. KCOTs associated with GGS are more common in the mandible (69%) than the maxilla (31%) [14].

There are two methods for the management of keratocystic odontogenic tumors: 1) conservative treatment, 2) aggressive treatment. [15]. In young patients (children), conservative treatment approaches should be always considered first because an aggressive treatment can cause unfavorable effects on the development of the involved jaw, teeth development, and the eruption process [16]. Furthermore, it was found that marsupialization followed by enucleation will result in the lowest recurrence rate in comparison to the other conservative treatment modalities [17]. According to Pogrel, marsupialization is a technique that involves altering the cyst to a pouch. Subsequently, the lesion will be decompressed [18].

To confirm the diagnosis, histopathological examination of the removed tissue should be done. In this case, the microscopic analysis of the two cystic lesions involving the molar-ramus region of the mandible confirmed the diagnosis of KCOT. Both of them were treated conservatively, the right KCOT lesion was enucleated while the left was treated using marsupialization, which will be followed by enucleation. In addition, the maxillary lesion was diagnosed as a dentigerous cyst.

It has been reported that the KCOT recurrence rate after the removal is high and ranges from 12% to 62.5% [19]. To decrease the secondary morbidities after the treatment, patients with KCOT should be followed and observed carefully by radiographic imaging, especially during the first year [20]. 

## Conclusions

Gorlin-Goltz syndrome is an autosomal dominant heredity disorder with multiple diagnostic criteria. The presented case confirms the idea that dentists have an important role in the early diagnosis; however, a multidisciplinary team is required for the management of this syndrome. Also, regular follow‐up by multi-specialists should be carried out for monitoring these patients.

## References

[1] Casaroto AR, Loures DC, Moreschi E, Veltrini VC, Trento CL, Gottardo VD, Lara VS (2011). Early diagnosis of Gorlin-Goltz syndrome: case report. Head Face Med.

[2] Jawa DS, Sircar K, Somani R, Grover N, Jaidka S, Singh S (2009). Gorlin-Goltz syndrome. J Oral Maxillofac. Pathol.

[3] Amezaga AOG, Arregui OG, Nuño SZ, Sagredo AA, Urizar JMA (2008). Gorlin-Goltz syndrome: clinicopathologic aspects. Med Oral Patol Oral Cir Bucal.

[4] Anderson DE, Taylor WB, Falls HF, Davidson RT (1967). The nevoid basal cell carcinoma syndrome. Am J Hum Genet.

[5] Gorlin RJ, Goltz RW (1960). Multiple nevoid basal-cell epithelioma, jaw cysts and bifid rib: a syndrome. N Engl J Med.

[6] Gorlin RJ (1999). Nevoid basal cell carcinoma (Gorlin) syndrome: unanswered issues. J Lab Clin Med.

[7] Gorlin RJ (1995). Nevoid basal cell carcinoma syndrome. Dermatol Clin.

[8] Manfredi M, Vescovi P, Bonanini M, Porter S (2004). Nevoid basal cell carcinoma syndrome: a review of the literature. Int J Oral Maxillofac Surg.

[9] Kimonis VE, Goldstein AM, Pastakia B (1997). Clinical manifestations in 105 persons with nevoid basal cell carcinoma syndrome. Am J Med Genet.

[10] Evans DG, Ladusans EJ, Rimmer S, Burnell LD, Thakker N, Farndon PA (1993). Complications of the naevoid basal cell carcinoma syndrome: results of a population based study. J Med Genet.

[11] Nilesh K, Tewary S, Zope S, Patel J, Vande A (2017). Dental, dermatological and radiographic findings in a case of Gorlin-Goltz Syndrome: report and review. Pan Afr Med J.

[12] Dowling PA, Fleming P, Saunders ID, Gorlin RJ, Napier SS (2000). Odontogenic keratocysts in a 5-year-old: Initial manifestations of nevoid basal cell carcinoma syndrome. Pediatr Dent.

[13] Sun LS, Li XF, Li TJ (2008). PTCH1 and SMO gene alterations in keratocystic odontogenic tumors. J Dent Res.

[14] Woolgar JA, Pippin JW, Browne RM (1987). The odontogenic keratocyst and its occurrence in the nevoid basal cell carcinoma syndrome. Oral Surg Oral Med Oral Pathol.

[15] Kolokythas A, Fernandes RP, Pazoki A, Ord RA (2007). Odontogenic keratocyst: to decompress or not to decompress? A comparative study of decompression and enucleation versus resection/peripheral ostectomy. J Oral Maxillofac Surg.

[16] Lench NJ, Telford EA, High AS, Markham AF, Wicking C, Wainwright BJ (1997). Characterization of human patched germ line mutations in naevoid basal cell carcinoma syndrome. Hum Genet.

[17] Nakamura N, Mitsuyasu T, Mitsuyasu Y, Taketomi T, Higuchi Y, Ohishi M (2002). Marsupialization for odontogenic keratocysts: Long-term follow-up analysis of the effects and changes in growth characteristics. Oral Surg Oral Med Oral Pathol Oral Radiol Endod.

[18] Pogrel MA (2005). Treatment of keratocysts the case for decompression and marsupialization. J Oral Maxillofac Surg.

[19] Oikarinen VJ (1990). Keratocyst recurrences at intervals of more than 10 years: case reports. Br J Oral Maxillofac Surg.

[20] Kuroyanagi N, Sakuma H, Miyabe S (2009). Prognostic factors for keratocystic odontogenic tumor (odontogenic keratocyst): analysis of clinico-pathologic and immunohistochemical findings in cysts treated by enucleation. J Oral Pathol Med.

